# Accurate sequence variant genotyping in cattle using variation-aware genome graphs

**DOI:** 10.1186/s12711-019-0462-x

**Published:** 2019-05-15

**Authors:** Danang Crysnanto, Christine Wurmser, Hubert Pausch

**Affiliations:** 1 Animal Genomics, ETH Zurich, Zurich, Switzerland; 2 Chair of Animal Breeding, TU München, Freising, Germany

## Abstract

**Background:**

Genotyping of sequence variants typically involves, as a first step, the alignment of sequencing reads to a linear reference genome. Because a linear reference genome represents only a small fraction of all the DNA sequence variation within a species, reference allele bias may occur at highly polymorphic or divergent regions of the genome. Graph-based methods facilitate the comparison of sequencing reads to a variation-aware genome graph, which incorporates a collection of non-redundant DNA sequences that segregate within a species. We compared the accuracy and sensitivity of graph-based sequence variant genotyping using the *Graphtyper* software to two widely-used methods, i.e., *GATK* and *SAMtools*, which rely on linear reference genomes using whole-genome sequencing data from 49 Original Braunvieh cattle.

**Results:**

We discovered 21,140,196, 20,262,913, and 20,668,459 polymorphic sites using *GATK*, *Graphtyper,* and *SAMtools*, respectively. Comparisons between sequence variant genotypes and microarray-derived genotypes showed that *Graphtyper* outperformed both *GATK* and *SAMtools* in terms of genotype concordance, non-reference sensitivity, and non-reference discrepancy. The sequence variant genotypes that were obtained using *Graphtyper* had the smallest number of Mendelian inconsistencies between sequence-derived single nucleotide polymorphisms and indels in nine sire-son pairs. Genotype phasing and imputation using the *Beagle* software improved the quality of the sequence variant genotypes for all the tools evaluated, particularly for animals that were sequenced at low coverage. Following imputation, the concordance between sequence- and microarray-derived genotypes was almost identical for the three methods evaluated, i.e., 99.32, 99.46, and 99.24% for *GATK*, *Graphtyper,* and *SAMtools,* respectively. Variant filtration based on commonly used criteria improved genotype concordance slightly but it also decreased sensitivity. *Graphtyper* required considerably more computing resources than *SAMtools* but less than *GATK*.

**Conclusions:**

Sequence variant genotyping using *Graphtyper* is accurate, sensitive and computationally feasible in cattle. Graph-based methods enable sequence variant genotyping from variation-aware reference genomes that may incorporate cohort-specific sequence variants, which is not possible with the current implementation of state-of-the-art methods that rely on linear reference genomes.

**Electronic supplementary material:**

The online version of this article (10.1186/s12711-019-0462-x) contains supplementary material, which is available to authorized users.

## Background

The sequencing of important ancestors of many cattle breeds revealed millions of sequence variants that are polymorphic in dairy and beef populations [1–4]. In order to compile an exhaustive catalog of polymorphic sites that segregate in *Bos taurus*, the 1000 Bull Genomes consortium was established [5, 6]. The 1000 Bull Genomes project imputation reference panel facilitates the inference of sequence variant genotypes for large cohorts of genotyped animals, thus enabling genomic investigations at the nucleotide level [5, 7–9].

Sequence variant discovery and genotyping typically involve two successive steps [10–13]. First, raw sequencing data are generated, trimmed and filtered to remove adapter sequences and bases with low sequencing quality, and then aligned to a linear reference genome using, e.g., *Bowtie* [14] or the Burrows-Wheeler Alignment (*BWA)* software [15]. Second, the aligned reads are compared to the nucleotide sequence of a reference genome in order to discover and genotype polymorphic sites using, e.g., *SAMtools* [16] or the *Genome Analysis Toolkit* (*GATK)* [17–19]. Variant discovery may be performed either in a single- or multi-sample mode. The accuracy (i.e., ability to correctly genotype sequence variants) and sensitivity (i.e., ability to detect true sequence variants) of sequence variant discovery is higher using multi-sample than single-sample approaches particularly when the sequencing depth is low [20–24]. However, genotyping sequence variants from multiple samples simultaneously is a computationally intensive task, particularly when the sequenced cohort is large and diverse and had been sequenced at high coverage [19]. The multi-sample sequence variant genotyping approach that is implemented in the *SAMtools* software has to be restarted for the entire cohort, once new samples are added. *GATK* implements two different approaches to multi-sample variant discovery, i.e., the *UnifiedGenotyper* and *HaplotypeCaller* modules, with the latter relying on intermediate files in gVCF format that include probabilistic data on variant and non-variant sites for each sequenced sample. Applying the *HaplotypeCaller* module allows to separate variant discovery within samples from the estimation of genotype likelihoods across samples. Once new samples are added to an existing cohort, only the latter needs to be performed for the entire cohort, thus enabling computationally efficient parallelization of sequence variant genotyping in a large number of samples.

Sequence variant genotyping approaches that rely on alignments to a linear reference genome have limitations for variant discovery, because a haploid reference sequence does not reflect variation within a species. As a result, read alignments may be erroneous particularly at genomic regions that differ substantially between the sequenced individual and the reference sequence, thus introducing reference allele bias, flawed genotypes, and false-positive variant discovery around indels [25–27]. Aligning reads to population- or breed-specific reference genomes may overcome most of these limitations [28–30]. However, considering multiple (population-specific) linear reference genomes with distinct genomic coordinates complicates the biological interpretation and annotation of sequence variant genotypes across populations [31].

Genome graph-based methods consider non-linear reference sequences for variant discovery [31–35]. A variation-aware genome graph may incorporate distinct (population-specific) reference sequences and known sequence variants. Recently, the *Graphtyper* software has been developed in order to facilitate sequence variant discovery from a genome graph that has been constructed and iteratively augmented using variants from the sequenced cohort [32]. To date, sequence variant genotyping using variation-aware genome graphs has not been evaluated in cattle.

An unbiased evaluation of the accuracy and sensitivity of sequence variant genotyping is possible when high confidence sequence variants and genotypes that were detected using genotyping technologies and algorithms different from those to be evaluated are available [36]. For species for which such a resource is not available, the accuracy of sequence variant genotyping may be evaluated by comparing sequence variant genotypes to microarray-derived genotypes (e.g., [2, 24]). Due to the ascertainment bias in single nucleotide polymorphism (SNP) chip data, this comparison may overestimate the accuracy of sequence variant discovery particularly at variants that are either rare or located in less accessible genomic regions [37, 38].

In this study, we compared sequence variant discovery and genotyping from a variation-aware genome graph using *Graphtyper* to two state-of-the-art methods (*GATK* and *SAMtools*) that rely on linear reference genomes from 49 Original Braunvieh cattle. We compared sequence variant genotypes to microarray-derived genotypes in order to assess accuracy and sensitivity of sequence variant genotyping for each of the three methods evaluated.

## Methods

### Selection of animals

We selected 49 Original Braunvieh (OB) bulls that were either frequently used in artificial insemination or explained a large fraction of the genetic diversity of the active breeding population. Semen straws of the bulls were purchased from an artificial insemination center and DNA was prepared following standard DNA extraction protocols.

### Sequencing data pre-processing

All samples were sequenced on either an Illumina HiSeq 2500 (30 animals) or an Illumina HiSeq 4000 (19 animals) sequencer using 150 bp paired-end sequencing libraries with insert sizes ranging from 400 to 450 bp. Quality control (removal of adapter sequences and bases with low quality) of the raw sequencing data was carried out using the *fastp* software (version 0.19.4) with default parameters [39]. The filtered reads were mapped to the UMD3.1 version of the bovine reference genome [40] using *BWA mem* (version 0.7.12) [15] with option-M to mark shorter split hits as secondary alignments, default parameters were applied in all other steps. Optical and PCR duplicates were marked using *Samblaster* (version 0.1.24) [41]. The output of *Samblaster* was converted into BAM format using *SAMtools view* (version 1.3) [16], and subsequently coordinate-sorted using *Sambamba* (version 0.6.6) [42]. We used the *GATK* (version 3.8) *RealignerTargetCreator* and *IndelRealigner* modules to realign reads around indels. The realigned BAM files served as input for *GATK* base quality score recalibration using 102,092,638 unique positions from the Illumina BovineHD SNP chip and Bovine dbSNP version 150, as known variants. The *mosdepth* software (version 0.2.2) [43] was used to extract the number of reads that covered a genomic position.

### Sequence variant discovery

We followed the best practice guidelines recommended for variant discovery and genotyping using *GATK* (version 4.0.6) with default parameters for all commands [17, 18, 24]. First, genotype likelihoods were calculated separately for each sequenced animal using *GATK HaplotypeCaller* [19], which resulted in files in gVCF (genomic Variant Call Format) format for each sample [44]. The gVCF files from the 49 samples were consolidated using *GATK GenomicsDBImport*. Subsequently, *GATK GenotypeGVCFs* was applied to genotype polymorphic sequence variants for all samples simultaneously.

*Graphtyper* (version 1.3) was run in a multi-sample mode as recommended in Eggertsson et al. [32]. Because the original implementation of *Graphtyper* is limited to the analysis of the human chromosome complement, we cloned the *Graphtyper GitHub* repository (https://github.com/DecodeGenetics/graphtyper), modified the source code to allow analysis of the cattle chromosome complement, and compiled the program from the modified source code (see Additional file 1). The *Graphtyper* workflow consisted of four steps that were executed successively. First, sequence variants were identified from the read alignments that were produced using *BWA mem* (see above). Second, these cohort-specific variants were used to augment the UMD3.1 reference genome and construct the variation-aware genome graph. Third, the sequencing reads were locally realigned against the variation-aware graph. A clean variation graph was produced by removing unobserved haplotypes paths from the raw graph. In the final step, genotypes were identified from the realigned reads in the clean graph. The *Graphtyper* pipeline was run in segments of 1 million bp and whenever the program failed to genotype variants for a particular segment either because it ran out of memory or exceeded the allocated runtime of 12 h, the interval was subdivided into smaller segments (10 kb).

Our implementation of *SAMtools mpileup* (version 1.8) [45] was run in a multi-sample mode to calculate genotype likelihoods from the aligned reads for all samples simultaneously. The parameters -*E* and -*t* were used to recalculate (and apply) base alignment quality and produce per-sample genotype annotations, respectively. Next, the estimated genotype likelihoods were converted into genotypes using *BCFtools call* using the -*v* and -*m* flags to output variable sites only, and permit sites to have more than two alternative alleles, respectively.

We implemented all pipelines using *Snakemake* (version 5.2.0) [46]. The scripts for the pipelines are available via *GitHub* repository (https://github.com/danangcrysnanto/Graph-genotyping-paper-pipelines).

### Sequence variant filtering and genotype refinement

The *GATK VariantFiltration* module was used to parse and filter the raw VCF files. Quality control on the raw sequencing variants and genotypes was applied according to guidelines that were recommended for each variant caller. Variants that were identified using *GATK* were retained if they met the following criteria: QualByDepth (QD) > 2.0, FisherStrand < 60.0, RMSMappingQuality (MQ) > 40.0, MappingQualityRankSumTest (MQRankSum) > 12.5, ReadPosRankSumTest (ReadPosRankSum) > -8.0, SOR < 3.0 (SNPs) and QD > 2.0, FS < 200.0, ReadPosRankSum > 20.0, SOR < 10.0 (indels). For the variants identified using *SAMtools*, the thresholds that have been applied in the 1000 Bull Genomes project [5] were considered to remove variants with indication of low quality. Variants were retained if they met the following criteria: QUAL > 20, MQ > 30, ReadDepth (DP) > 10, DP < median(DP) + 3 * mean(DP). Moreover, SNPs were removed from the data if they had the same positions as the starting position of an indel. The output of *Graphtyper* was filtered so that it included only variants that met the criteria recommended by Eggertsson et al. [32]: ABHet < 0.0 | ABHet > 0.33, ABHom < 0.0 | ABHom > 0.97, MaxAASR > 0.4, and MQ > 30.

We used *Beagle* (version 4.1) [47] to improve the raw sequence variant genotype quality and impute missing genotypes. The genotype likelihood (*gl*) mode of *Beagle* was applied to infer missing and modify existing genotypes based on the phred-scaled likelihoods (*PL*) of all other non-missing genotypes of the 49 Original Braunvieh animals in our study.

### Evaluation of sequence variant genotyping

To ensure consistent variant representation across the different sequence variant genotyping methods evaluated, we applied the *vt normalize* software (version 0.5) [48]. Normalized variants are parsimonious (i.e., represented by as few nucleotides as possible) and left aligned [48]. The number of variants detected and transition to transversion (Ti/Tv) ratios were calculated using *vt peek* [48] and *BCFtools stats* [45]. The intersection of variants that were common to the evaluated tools was calculated and visualized using *BCFtools isec* [45] and the UpSet R package [49], respectively.

Mendelian inconsistencies were calculated as the proportion of variants showing opposing homozygous genotypes in nine parent–offspring pairs that were included in the 49 sequenced animals. For this comparison, we considered only the sites for which the genotypes of both sire and son were not missing.

All 49 sequenced cattle were also genotyped using either the Illumina BovineHD (N = 29) or the BovineSNP50 (N = 20) Bead chip that comprise 777,962 and 54,001 SNPs, respectively. The average genotyping rate at autosomal SNPs was 98.91%. In order to assess the quality of sequence variant genotyping, the genotypes detected by the different variant calling methods were compared to the array-called genotypes in terms of genotype concordance, non-reference sensitivity and non-reference discrepancy [24, 50], and for more details on the metrics (see Additional file 2). Non-parametric Kruskal–Wallis tests followed by pairwise Wilcoxon signed-rank tests were applied to determine if any of the three metrics differed significantly between the three tools evaluated.

### Computing environment and statistical analysis

All computations were performed on the ETH Zurich Leonhard Open Cluster with access to multiple nodes equipped with 18 cores Intel Xeon E5-2697v4 processors (base frequency rated at 2.3 GHz) and 128 GB of random-access memory. Unless otherwise stated, the R (version 3.3.3) software environment [51] was used for statistical analyses and ggplot2 (version 3.0.0) [52] was used for data visualisation.

## Results

**Fig. 1:**
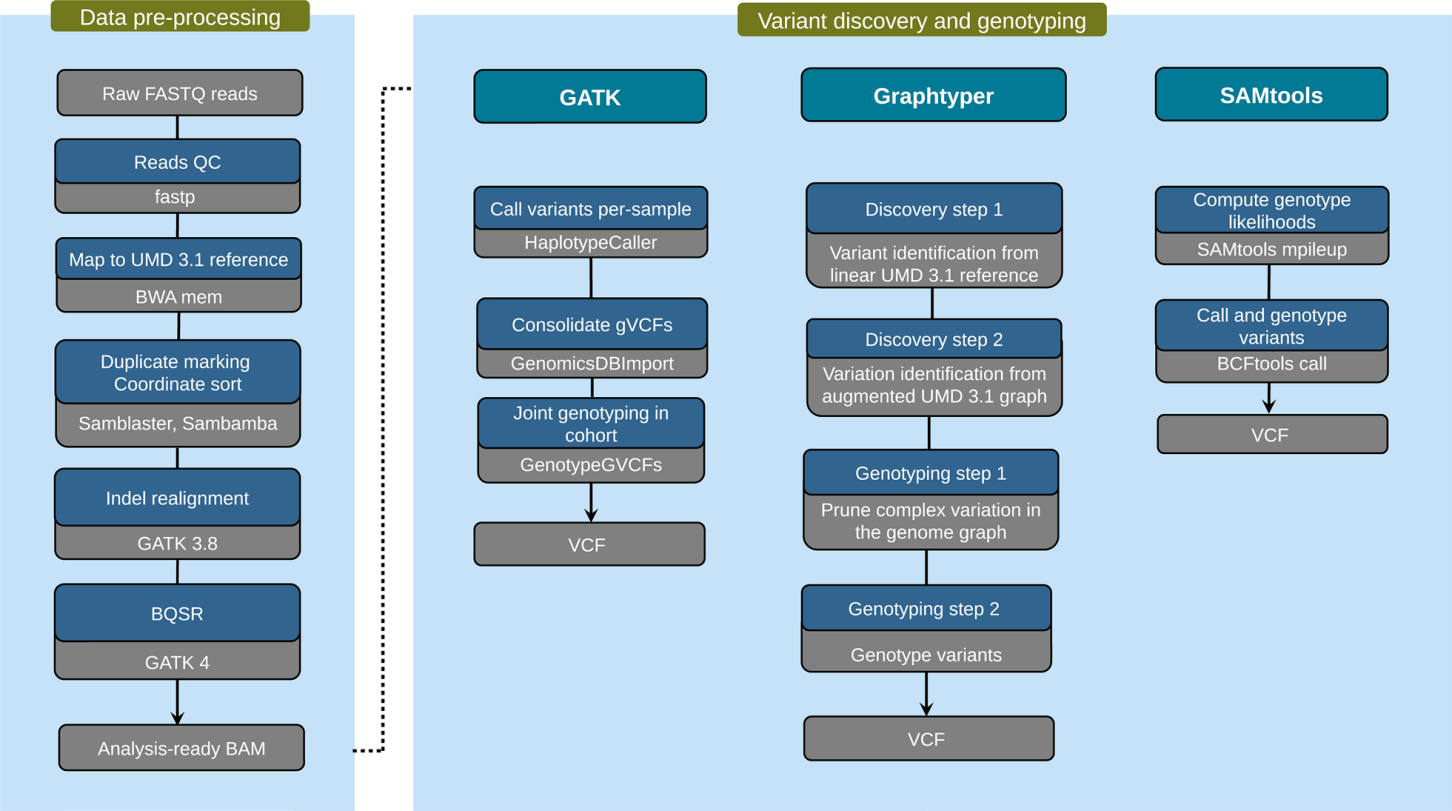
Schematic representation of the three sequence variant discovery and genotyping methods evaluated. According to the best practice recommendations for sequence variant discovery using *GATK*, the VQSR module should be applied to distinguish between true and false positive variants. Because this approach requires a truth set of variants, which is not (publicly) available for cattle, the VQSR module was not considered in our evaluation

Following quality control (removal of adapter sequences and low-quality bases), we aligned more than 13 billion paired-end reads (2 × 125 and 2 × 150 bp) from 49 Original Braunvieh cattle to the UMD3.1 assembly of the bovine genome. On average, 98.44% (91.06–99.59%) of the reads mapped to the reference genome and 4.26% (2.0–10.91%) of these were flagged as duplicates and not considered for further analyses. Sequencing depth ranged from 6.00 to 37.78 with an average depth per animal of 12.75 and was above 12-fold for 31 samples. Although the realignment of sequencing reads around indels is no longer required when sequence variants are genotyped using the latest version of *GATK* (v 4), it is still recommended to improve the genotyping of indels by using *SAMtools*. To ensure a fair comparison of the three tools evaluated, we realigned the reads around indels on all BAM files and used the re-aligned files as a starting point for our comparisons (Fig. 1). The sequencing read data of 49 cattle were deposited at European Nucleotide Archive (ENA) (http://www.ebi.ac.uk/ena) under primary accession PRJEB28191.

### Sequence variant discovery and genotyping

Polymorphic sites (SNPs, indels) were discovered and genotyped in the 49 animals using either *GATK* (version 4), *Graphtyper* (version 1.3) or *SAMtools* (version 1.8). All software programs were run using default parameters and workflow descriptions for variant discovery (Fig. 1 and also see Methods). Only autosomal sequence variants were considered to evaluate the accuracy and sensitivity of sequence variant genotyping. Because variant filtering has a strong impact on the accuracy and sensitivity of sequence variant genotyping [53, 54], we evaluated both the raw variants that were detected using default parameters for variant discovery (Fig. 1) and variants that remained after applying filtering criteria that are commonly used but may differ slightly between different software tools. Note that *GATK* was run by using the suggested filtering parameters, when application of *Variant Quality Score Recalibration* (VQSR) is not possible.

Using default parameters for variant discovery for each of the software programs evaluated, 21,140,196, 20,262,913, and 20,668,459 polymorphic sites were discovered using *GATK, Graphtyper* and *SAMtools*, respectively (Table 1**)**. The vast majority (86.79, 89.42 and 85.11%) of the detected variants were biallelic SNPs. Of the 18,594,182, 18,120,724 and 17,592,038 SNPs detected using *GATK*, *Graphtyper* and *SAMtools*, respectively, 7.46, 8.31 and 5.02% were novel, i.e., they were not among the 102,091,847 polymorphic sites of the most recent version (150) of the Bovine dbSNP database. The Ti/Tv ratio of the detected SNPs was equal to 2.09, 2.07 and 2.05 using *GATK*, *Graphtyper* and *SAMtools*, respectively. Using *GATK* revealed four times more multiallelic SNPs (246,220) than either *SAMtools* or *Graphtyper*.

**Table 1:** Number of different types of autosomal sequence variants detected in 49 Original Braunvieh cattle using three sequence variant genotyping methods (Full) and subsequent variant filtration based on commonly used criteria (Filtered)

	Full			Filtered		
	*GATK*	*Graphtyper*	*SAMtools*	*GATK*	*Graphtyper*	*SAMtools*
Variants	21,140,196	20,262,913	20,668,459	19,761,679	17,679,155	18,871,549
SNPs	18,594,182	18,120,724	17,592,038	17,248,593	15,777,446	16,272,917
Not in dbSNP	1,387,781	1,505,586	882,575	867,838	564,326	570,901
Biallelic	18,347,962	18,053,396	17,528,249	17,111,806	15,730,153	16,218,714
Multi-allelic	246,220	67,328	63,789	136,787	47,293	54,203
Ti/Tv ratio	2.09	2.07	2.05	2.17	2.18	2.16
SNP array (%)						
BovineHD	99.46	99.61	99.32	99.21	98.79	98.85
Bovine SNP50	99.14	99.26	99.12	98.91	98.87	98.90
Indels	2,478,489	2,044,585	3,076,421	2,445,766	1,826,808	2,598,632
Not in dbSNP	663,831	596,137	1,279,162	639,219	456,752	979,291
Biallelic	2,166,352	1,753,391	2,704,413	2,133,840	1,571,195	2,310,386
Multi-allelic	312,137	291,194	372,008	311,926	255,613	288,246
Insertion/Deletion	0.88	0.88	1	0.88	0.88	0.99
Complex variation	67,525	97,604	0	67,320	74,901	0

We identified 2,478,489, 2,044,585, and 3,076,421 indels using *GATK*, *Graphtyper*, and *SAMtools*, respectively, and 26.78%, 29.15%, and 41.75% of them were novel. *SAMtools* revealed the largest number and highest proportion (14.9%) of indels. Between 12 and 14% of the detected indels were multiallelic. While *Graphtyper* and *GATK* identified more (12%) deletions than insertions, the proportions were almost the same using *SAMtools*.

On average, each Original Braunvieh cattle carried between 7 and 8 million variants that differed from the UMD3.1 reference genome. Of these, between 2.4 and 2.6 million SNPs were homozygous for the alternate allele, between 3.8 and 4.7 million SNPs were heterozygous and between 0.7 and 1 million were indels (Table 2).

**Table 2:** Average number of autosomal variants identified per animal using three sequence variant genotyping methods

	Full			Filtered		
	*GATK*	*Graphtyper*	*SAMtools*	*GATK*	*Graphtyper*	*SAMtools*
Total biallelic SNPs	6,324,455	7,384,058	6,617,948	6,105,674	6,533,711	6,564,229
Heterozygous	3,890,351	4,758,297	4,187,882	3,744,336	4,074,011	4,147,033
Homozygous ALT	2,434,104	2,625,761	2,430,066	2,361,338	2,459,700	2,417,196
Ti/Tv	2.17	2.13	2.11	2.20	2.14	2.13
Total biallelic indels	693,697	767,261	1,007,420	691,765	697,637	960,218
Heterozygous	390,495	441,172	616,981	388,622	391,856	593,417
Homozygous ALT	303,202	326,089	390,439	303,143	305,781	366,801
Singletons	49,166	23,406	32,810	41,408	17,999	32,398

The number of variants is presented for the three tools evaluated before (Full) and after (Filtered) applying recommended filters to identify and exclude low quality variants

**Fig. 2:**
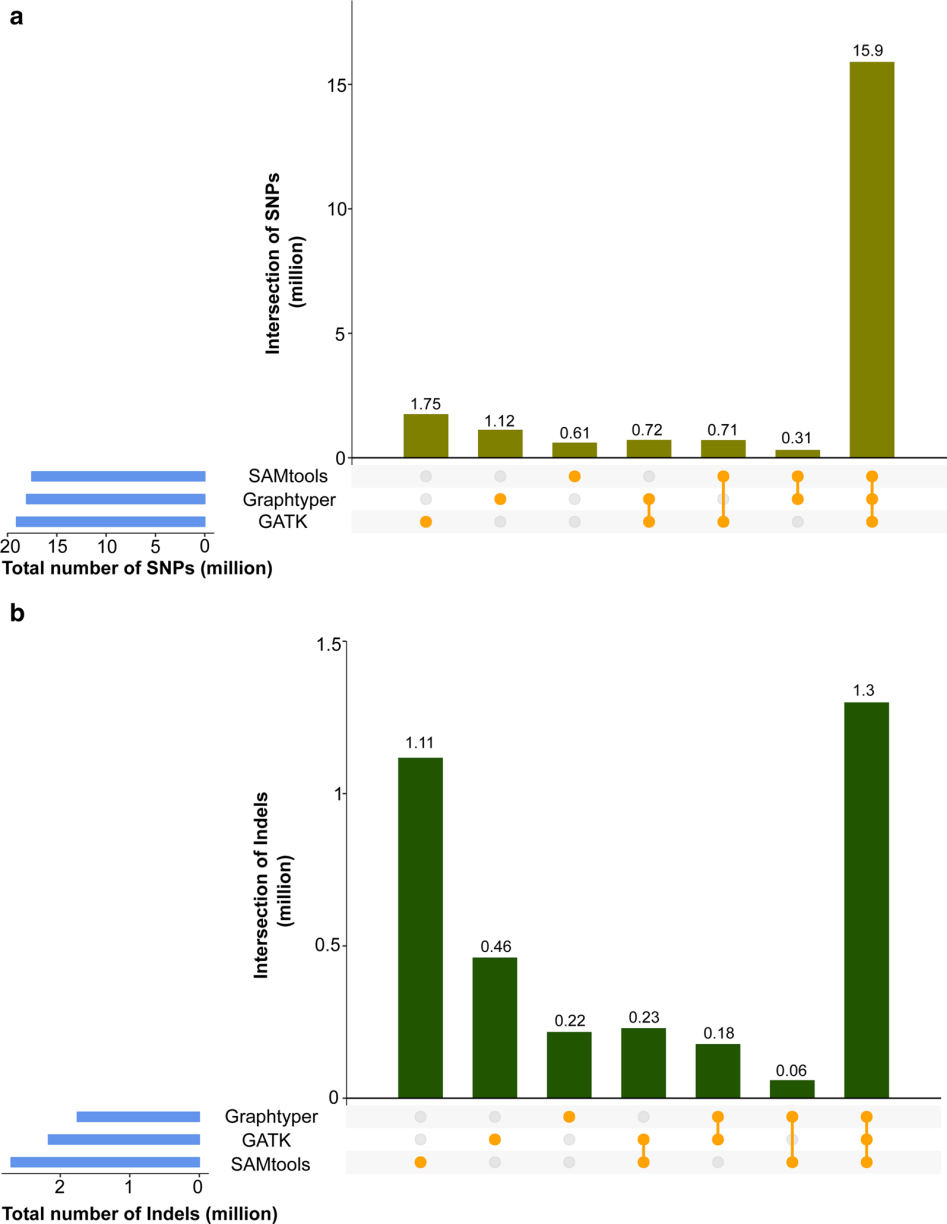
Number of biallelic SNPs (**a**) and indels (**b**) identified in 49 Original Braunvieh cattle using three sequence variant genotyping methods. Blue horizontal bars represent the total number of sites discovered for each method. Vertical bars indicate private and common variants detected by the methods evaluated

An intersection of 15,901,526 biallelic SNPs was common to all sequence-variant discovery tools evaluated (Fig. 2a), i.e., between 85.51 and 90.39% of the detected SNPs of each tool, and 466,029 (2.93%, Ti/Tv: 1.81) of them were novel, i.e., they were not present in dbSNP 150. The Ti/Tv-ratio of the common SNPs was 2.22. *SAMtools* had the largest number of SNPs in common with the other two tools (90.39%). The number of private SNPs, i.e., SNPs that were detected by one but not the other tools was largest for *GATK* and smallest for *Graphtyper*.

In total, 1,299,467 biallelic indels (Fig. 2b) were common to all evaluated tools and 98,931 (13.13%) of these were novel, i.e., they were not present in dbSNP 150. The intersection among the three tools was considerably smaller for indels than for SNPs. *Graphtyper* had the highest proportion of indels in common with the other tools (74.11%). *SAMtools* discovered the largest number (2,704,413) of biallelic indels and most of them (41.26%) were not detected using either *GATK* or *Graphtyper*. *GATK* (21.2%) and *Graphtyper* (12.38%) discovered fewer private indels than *SAMtools*.

### Sequence variant genotyping using *Graphtyper* is accurate

The 49 sequenced animals were also genotyped using either the Illumina BovineHD or the Illumina BovineSNP50 Bead chip. Genotype concordance, non-reference sensitivity and non-reference discrepancy were calculated using array-called and sequence variant genotypes at corresponding positions. Genotype concordance is a measure of the proportion of variants that have identical genotypes on the microarray and in whole-genome sequencing data. Non-reference sensitivity is the proportion of microarray-derived variants that were also detected in the sequencing data. Non-reference discrepancy reflects the proportion of sequence variants that have genotypes that differ from the microarray-derived genotypes [for more details on how the different metrics were calculated (see Additional file 2)]. All metrics were calculated both for raw and filtered variants either before or after applying the algorithm implemented in the *Beagle* software for haplotype phasing and imputation.

In the raw data, the proportion of missing non-reference sites was 1.90%, 0.56%, and 0.47% using *GATK*, *Graphtyper,* and *SAMtools,* respectively. The genotype concordance between the sequence- and microarray-derived genotypes was higher (P < 0.005) when *Graphtyper* (97.72%) was used than when either *SAMtools* (97.68%) or *GATK* (95.99%) was used (Table 3). For the three tools evaluated, the genotype concordance was higher at homozygous than heterozygous sites, particularly in animals that were sequenced at low depth (see Additional file 3) In order to take the variable proportions of missing genotypes in the sequence variants into account, we calculated non-reference sensitivity and non-reference discrepancy. Non-reference sensitivity was almost identical using *Graphtyper* (98.26%) and *SAMtools* (98.21%). However, non-reference sensitivity was clearly lower using *GATK* (93.81%, *P* < 0.001). Non-reference discrepancy was lower using *Graphtyper* (3.53%) than using either *SAMtools* (3.6%, *P* = 0.003) or *GATK* (6.35%, *P* < 0.001).

**Table 3:** Comparisons between array-called and sequence variant genotypes

	Genotype concordance				Non-reference sensitivity				Non-reference discrepancy			
	Full		Filtered		Full		Filtered		Full		Filtered	
	Raw	Imp	Raw	Imp	Raw	Imp	Raw	Imp	Raw	Imp	Raw	Imp
*GATK*	95.99***	99.32***	96.02***	99.39***	93.81***	*99.36*	93.67***	*99.15*	6.35***	1.05***	6.3***	0.95***
*Graphtyper*	*97.71*	*99.46*	*97.75*	*99.52*	*98.26*	99.35	*97.91*	99.00***	*3.53*	*0.83*	*3.47*	*0.73*
*SAMtools*	97.68***	99.24***	97.7*	99.29***	98.21	99.35	97.53***	98.67***	3.6**	1.17***	3.56**	1.09***

Genotype concordance, non-reference sensitivity and non-reference discrepancy (in percentage) were calculated between the genotypes from the Bovine SNP Bead chip and sequence–derived genotypes for 49 Original Braunvieh cattle considering either the raw or imputed (Imp) sequence variant genotypes before (Full) and after (Filtered) variants were filtered based on commonly used criteria. Asterisks denote significant differences (*P ≤ 0.05, **P ≤ 0.01, ***P ≤ 0.001) with the best value (italic) for a respective parameter

Next, we analysed the proportion of opposing homozygous genotypes for SNPs and indels in nine sire-son pairs that were included among the sequenced animals (Table 4). We observed that SNPs that were discovered using either *Graphtyper* or *SAMtools* had almost a similar proportion of genotypes with Mendelian inconsistencies in the full and filtered datasets, whereas the values were two times higher using *GATK*. The proportion of opposing homozygous genotypes was higher for indels than SNPs for all the tools evaluated. However, in the full and filtered datasets, it was lower when *Graphtyper* was used than when either *GATK* or *SAMtools* was used. Using filtering parameters that are commonly applied for the three evaluated tools (see Methods), we excluded 1,378,517 (6.52%, Ti/Tv 1.24), 2,583,758 (12.75%, Ti/Tv 1.47) and 1,796,910 (8.69%, Ti/Tv 1.36) variants due to low mapping or genotyping quality from the *GATK*, *Graphtyper*, and *SAMtools* datasets, respectively. The genotype concordance between sequence- and microarray-derived genotypes was slightly higher for the filtered than the raw genotypes, but the non-reference sensitivity was lower for the filtered than the raw genotypes, which indicates that the filtering step also removed some true variant sites from the raw data (Table 3). The filtering step had almost no effect on the proportion of Mendelian inconsistencies detected in the nine sire-son pairs (Table 4).

**Table 4:** Proportions of opposing homozygous genotypes observed in nine sire-son pairs

	SNPs				Indels			
	Full		Filtered		Full		Filtered	
	Raw	Imp	Raw	Imp	Raw	Imp	Raw	Imp
*Bovine HD SNP array*	*0.001*							
*GATK*	0.73*	0.15*	0.72*	0.13*	0.98*	0.24*	0.99*	0.21*
*Graphtyper*	0.36	*0.11*	0.36	*0.11*	*0.54*	*0.13*	*0.54*	*0.13*
*SAMtools*	*0.33*	0.28*	*0.32*	0.25*	0.67	0.54*	0.61	0.57*

The ratio (in percentage) was calculated using autosomal sequence variants considering either the raw or imputed (Imp) sequence variant genotypes before (Full) and after (Filtered) variants were filtered based on commonly used criteria. Asterisks denote significant differences (*P ≤ 0.05, **P ≤ 0.01, ***P ≤ 0.001) with the best value (italic) for a respective parameter

### *Beagle* genotype refinement improved genotype quality

**Fig. 3:**
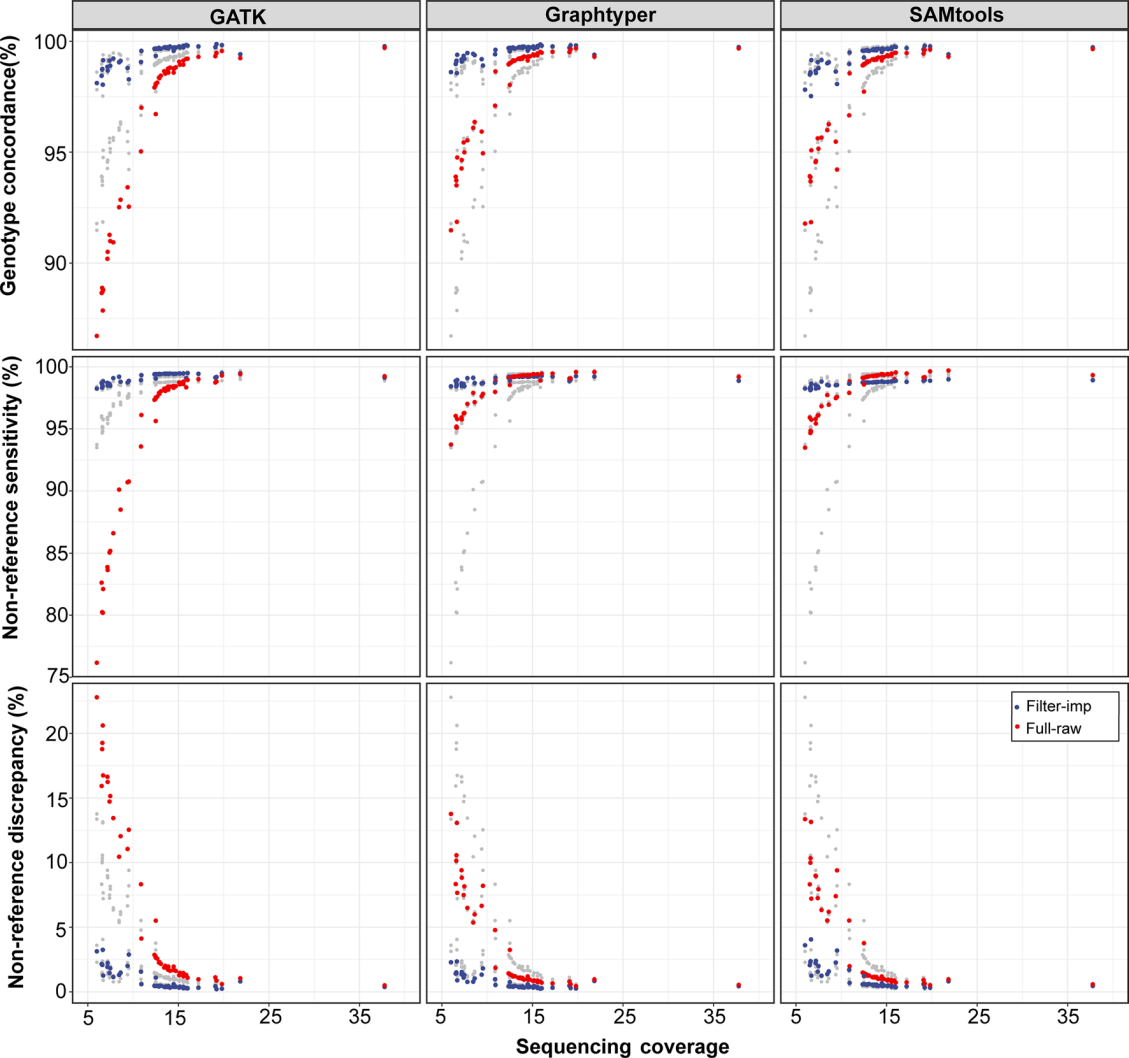
Accuracy and sensitivity of sequence variant genotyping at different sequencing depths. Genotype concordance, non-reference sensitivity and non-reference discrepancy were calculated for 49 Original Braunvieh cattle considering either raw (red) or filtered and imputed (blue) sequence variant genotypes. The grey points represent overlays of the results from the other methods

We used the *Beagle* software to refine the primary genotype calls and infer missing genotypes in the raw and filtered datasets. Following imputation, the quality of the sequence variant genotypes increased for all evaluated tools particularly for the individuals that had a sequencing coverage less than 12-fold (Fig. 3). The largest increase in the concordance metrics was observed for the sequence variants that were obtained using *GATK* (Tables 3 and 4). Following imputation, the variants identified using *Graphtyper* had a significantly higher quality (*P* < 0.05) for eight out of the ten metrics evaluated.

The quality of the sequence variant genotypes, particularly before *Beagle* genotype phasing and imputation, was influenced by the variable depth of coverage for the 49 sequenced samples of our study (Fig. 3). When we restricted the evaluations to 31 samples that had an average sequencing depth above 12-fold, the three tools performed almost identically (see Additional file 4). However, the performance of *Graphtyper* was significantly (*P* < 0.05) higher for 12 (out of the total 20) metrics than either that of *GATK* or *SAMtools*. When 18 samples with an average sequencing depth lower than 12-fold were considered, the differences observed in the three metrics were more pronounced between the three tools. In samples with a low sequencing coverage, *Graphtyper* performed significantly (*P* < 0.05) better than either *GATK* or *SAMtools* for all concordance metrics both before and after filtering and *Beagle* imputation, except for the non-reference sensitivity.

### Computing requirements

**Fig. 4:**
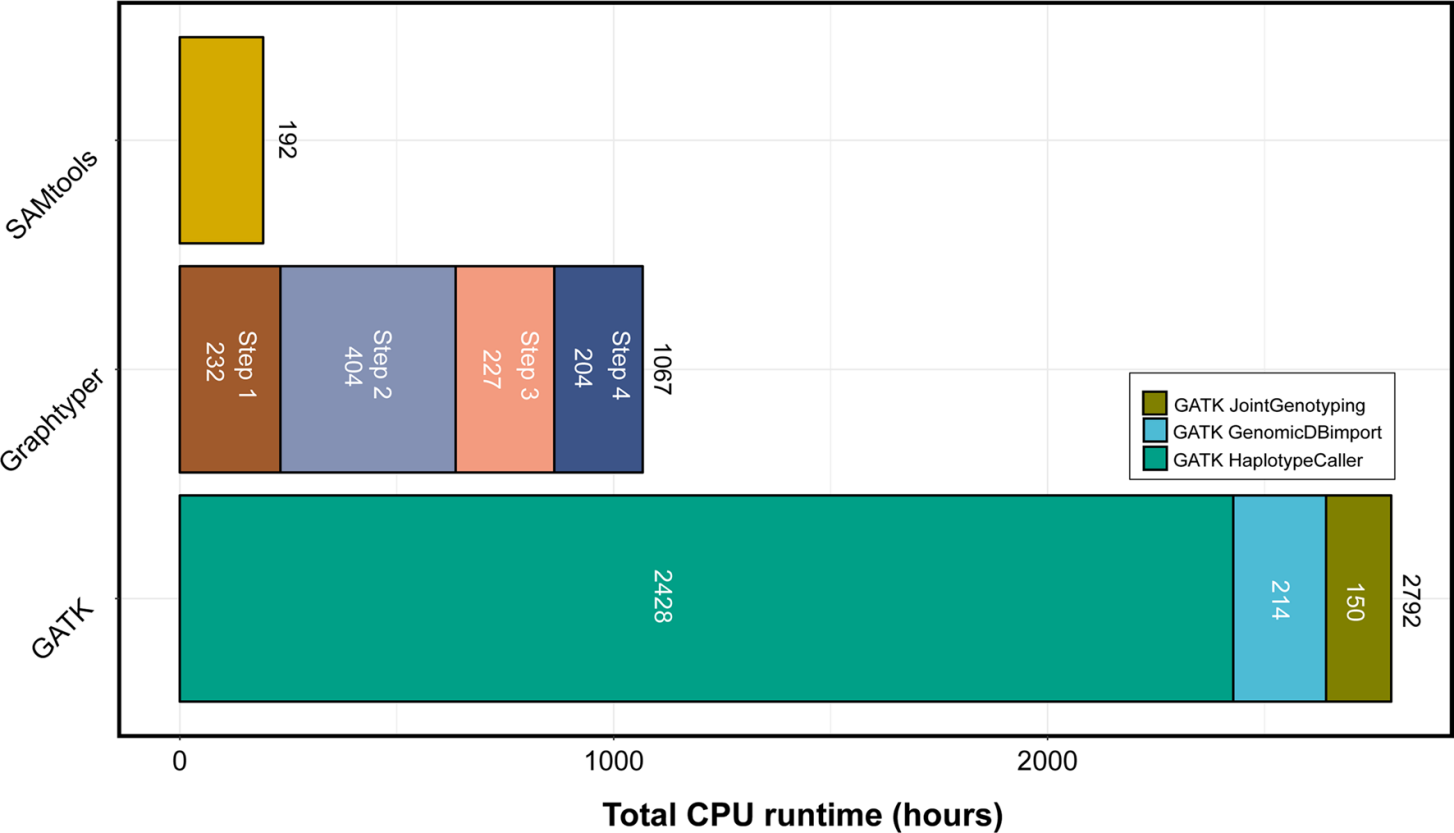
Computing time required to genotype all autosomal sequence variants in 49 Original Braunvieh cattle. The runtime of *GATK* and *Graphtyper* is shown for the different steps (see Fig. 1 for more details)

The multi-sample sequence variant genotyping pipelines that were implemented using either *GATK* or *SAMtools* were run separately for each chromosome in a single-threading mode. The *SAMtools mpileup* module took between 3.07 and 11.4 CPU hours and required between 0.12 and 0.25 gigabytes (GB) peak random-access memory (RAM) per chromosome. To genotype 20,668,459 sequence variants in 49 animals, *SAMtools* mpileup required 192 CPU hours (Fig. 4).

For *GATK*, we submitted 1421 parallel jobs of the *HaplotypeCaller* module (i.e., one job for each animal and chromosome) that required between 3.9 and 12.3 GB RAM and between 0.36 and 11 CPU hours to complete. To process 29 chromosomes in 49 samples, the *HaploytpeCaller* module required 2428 CPU hours. Subsequently, we ran the *GATK GenomicsDBImport* module, which required between 7.98 and 20.88 GB RAM and between 2.81 and 19.31 CPU hours per chromosome. *GATK Joint Genotyping* required between 4.33 and 17.32 GB of RAM and between 1.81 and 14.01 h per chromosome. To genotype 21,140,196 polymorphic sequence variants in 49 animals, the *GATK* pipeline required 2792 CPU hours (Fig. 4).

The *Graphtyper* pipeline including construction of the variation graph and genotyping of sequence variants was run in parallel for 2538 non-overlapping segments of 1 million bp as recommended by Eggertson et al. [32]. The peak RAM required by *Graphtyper* was between 1 and 3 GB per segment. Twelve segments, for which *Graphtyper* either ran out of memory or did not finish within the allocated time, were subdivided into smaller segments of 10 kb and subsequently re-run (Additional file 5). The genotyping of 20,262,913 polymorphic sites in 49 animals using our implementation of the *Graphtyper* pipeline required 1066 CPU hours (Fig. 4).

**Fig. 5:**
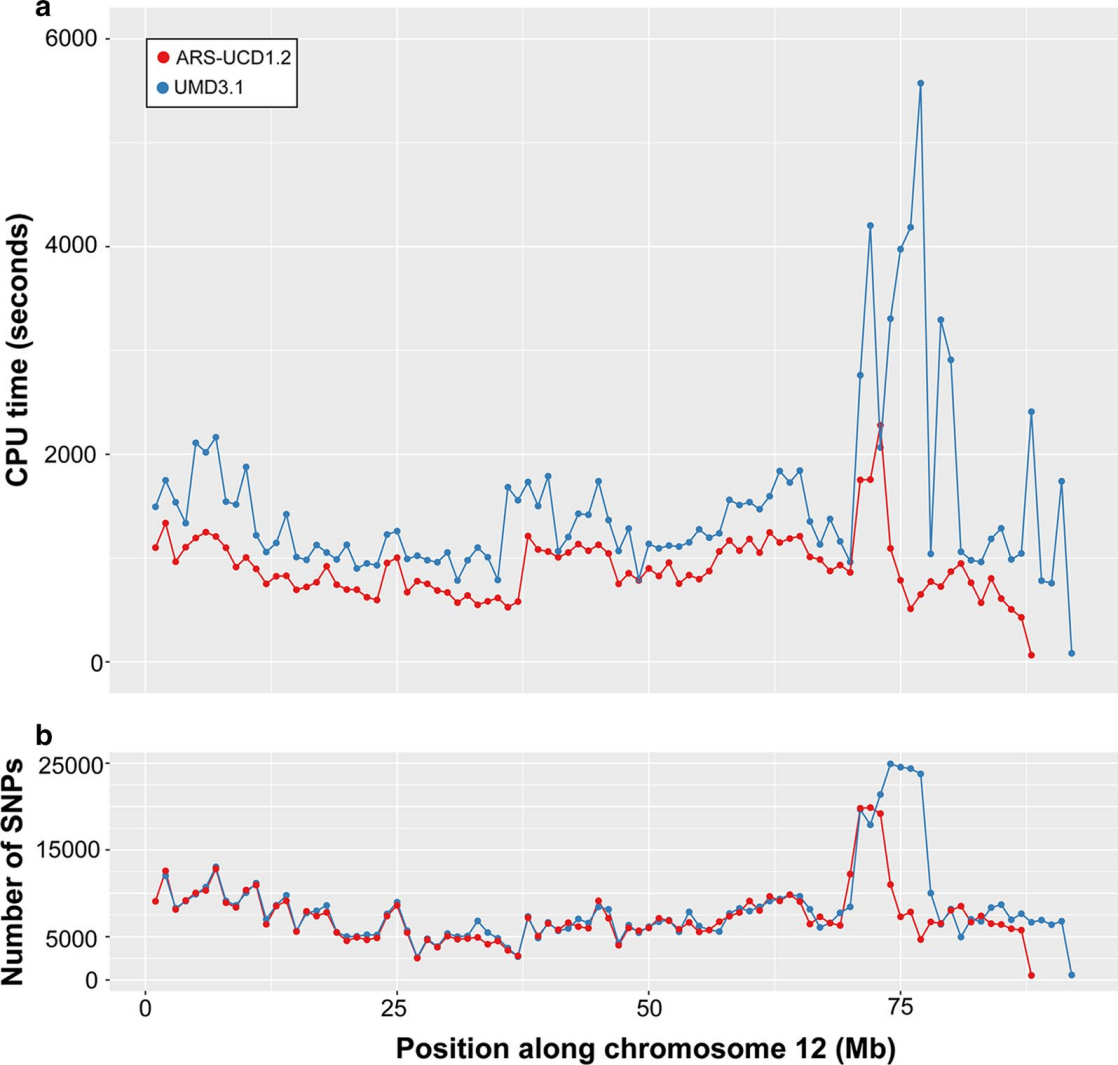
Sequence variant genotyping on chromosome 12 using Graphtyper. Computing time required (**a**) and number of variants discovered (**b**) for bovine chromosome 12 using *Graphtyper*. Each dot represents an interval of 1 million bp. Blue and red colours represent values for the UMD3.1 and ARS-UCD1.2 versions of the bovine assembly, respectively

The computing resources required by *SAMtools* and *GATK* increased linearly with chromosome length. The computing time required to genotype sequence variants was highly heterogeneous along the genome using *Graphtyper*. The CPU time for a 1-Mb segment ranged from 0.196 to 10.11 h, with an average CPU time of 0.42 h. We suspected that flaws in the reference genome might increase the complexity of the variation-aware graph and that the construction of the graph might benefit from an improved assembly. To test this hypothesis, we re-mapped the sequencing reads to the recently released new bovine reference genome (ARS-UCD1.2, https://www.ncbi.nlm.nih.gov/assembly/GCF_002263795.1) and repeated the graph-based sequence variant discovery. Indeed, we did observe a decrease in the computing time required to genotype polymorphic sites (particularly at chromosomes 12, 27 and 29) and a more uniform runtime along the genome, which possibly indicates that graph-based variant discovery in cattle will be faster and more accurate with highly contiguous reference sequences (Fig. 5).

## Discussion

We used either *GATK*, *Graphtyper*, or *SAMtools* to discover and genotype polymorphic sequence variants in whole-genome sequencing data of 49 Original Braunvieh cattle that were sequenced at between 6 and 38-fold genome coverage. Whereas *SAMtools* and *GATK* discover variants from a linear reference genome, *Graphtyper* locally realigns reads to a variation-aware reference graph that incorporates cohort-specific sequence variants [32]. Our graph-based variant discovery pipeline that is implemented by using the *Graphtyper* software used the existing bovine reference sequence to construct the genome graph. Subsequently, the graph was augmented with variants that were detected from linear alignments of the 49 Original Braunvieh cattle. The use of more sophisticated genome graph-based approaches that have been developed very recently facilitates the mapping of raw sequencing reads directly against a genome graph without the need to first align reads towards a linear reference genome [34]. Whereas genome graph-based variant discovery has been explored recently in mammalian-sized genomes [27, 31, 32, 35], our work is the first to apply graph-based sequence variant genotyping in cattle.

In order to evaluate graph-based variant discovery in cattle, we compared accuracy and sensitivity of *Graphtyper* to *GATK*, and *SAMtools* , i.e., two state-of-the-art methods on linear reference genomes that have been evaluated thoroughly in many species including cattle [2, 20]. We ran each tool with default parameters for variant discovery and applied commonly used or recommended filtration criteria. However, our evaluation of the software tools may suffer from ascertainment bias because we relied on SNPs that are included in bovine SNP arrays, i.e., they are located predominantly at genomic regions where variants are easy to identify [37, 38, 50]. Thus, the global accuracy and sensitivity of sequence variant discovery might be overestimated in our study. However, this ascertainment bias is unlikely to affect the relative performance of the methods evaluated.

In 49 Original Braunvieh cattle, sequence variant genotyping was more accurate using *Graphtyper* than either *GATK* or *SAMtools.* Differences in accuracy are small between the three tools, particularly when samples are sequenced at an average coverage higher than 12-fold (see Additional file 4). Yet, *Graphtyper* performed significantly better than *GATK* and *SAMtools* for samples sequenced at medium (> 12-fold) or low (< 12-fold) coverage indicating that genome graph-based variant discovery in cattle is accurate across a wide range of sequencing depths. *GATK* might perform better than observed in our study, when the VQSR module is applied to train the variant filtration algorithm on true and false variants [55]. However, to the best of our knowledge, the required sets of true and false variants are not available in cattle. An intersection of variants detected by different sequence variant genotyping software may be considered as a truth set (e.g., [56]) and compiling such a set is possible using the 49 samples from our study. However, a truth set that has been constructed from the data that are used for evaluation is likely to be depleted for variants that are difficult to discover in the target data set, thus preventing an unbiased evaluation of variant calling [36]. Variants from the 1000 Bull Genomes project [5, 6] could potentially serve as a truth/training set. However, variants from the 1000 Bull Genomes project were detected from short read sequencing data using either *GATK* or *SAMtools*, i.e., technologies and software that are part of our evaluation, thus precluding an unbiased comparison of variant discovery between *GATK*, *Graphtyper*, and *SAMtools* [36]. Vander Jagt et al. [57] showed in a subset of samples from the 1000 Bull Genomes project that *GATK* VQSR does not notably improve the concordance between sequence-derived and microarray-called genotypes compared to *GATK* hard filtering. Interestingly, the proportion of opposing homozygous genotypes in sire/offspring pairs was slightly higher in their study using *GATK* VQSR than *GATK* hard-filtering as used by the 1000 Bull Genomes project [57]. Applying *GATK* VQSR to the variants of our dataset corroborates the findings of Vander Jagt et al. [57] (see Additional file 6). Considering that the quality of the truth/training sets has a strong impact on the capabilities of VQSR (Additional file 6) and that high-confidence variants are currently not publicly available for cattle, we report *GATK* results using the recommended filtering parameters when VQSR is not possible.

Regardless of the method evaluated, we observed heterozygous under-calling in animals that are sequenced at low coverage, i.e., heterozygous variants were erroneously genotyped as homozygous due to an insufficient number of sequencing reads supporting the heterozygous genotype [10, 58–60]. In agreement with previous studies [2, 5], *Beagle* imputation improved genotype concordance and reduced heterozygous under-calling particularly in individuals that are sequenced at low coverage. After the imputation step, the genotype concordance, non-reference sensitivity, and non-reference discrepancy of the three tools were almost identical, which indicates that genotyping sequence variants from samples with a medium genome coverage is possible at high accuracy (at least for common variants in more accessible regions of the genome) using any of the three tools evaluated and subsequent *Beagle* error correction. While such conclusions have been drawn previously for *SAMtools* and *GATK* [2, 20], our findings demonstrate that the genotype likelihoods estimated from the *Graphtyper* software are also compatible with and benefit from the imputation algorithm implemented in the *Beagle* software. Considering that sequence data are enriched for rare variants that are more difficult to impute than common variants from SNP microarrays [61], the benefits from *Beagle* error correction might be overestimated in our study. An integration of phasing and imputation of missing genotypes directly in a graph-based variant genotyping approach would simplify sequence variant genotyping from variation-aware graphs [31, 62, 63]. Using *Graphtyper* for variant genotyping and *Beagle* for genotype refinement enabled us to genotype sequence variants in 49 Original Braunvieh cattle at a genotypic concordance of 99.52%, i.e., higher than previously achieved using either *GATK* or *SAMtools* for variant calling in cattle that are sequenced at a similar genome coverage [2–5, 20, 64]; this indicates that graph-based variant discovery might improve sequence variant genotyping. However, applying the filtering criteria that are recommended for *Graphtyper* [32] removed more variants from the *Graphtyper* (12.75%) than from either *GATK* (6.52%) or *SAMtools* (8.69%) datasets. It should be mentioned that *GATK* VQSR would remove considerably more variants from the *GATK* dataset than *GATK* hard filtering as applied in our study (see Additional file 6). Fine-tuning of the variant filtering parameters is necessary to further increase the accuracy and sensitivity of sequencing variant genotyping, particularly for *Graphtyper* [53, 54]. Moreover, the accuracy and sensitivity of graph-based variant discovery may be higher when known variants are considered for the initial construction of the variation graph [32]. Indeed, we observed a slight increase in genotype concordance (see Additional file 7) when we used *Graphtyper* to genotype sequence variants from a variation-aware genome-graph that incorporated bovine variants listed in dbSNP 150. However, additional research is required to prioritize a set of variants to augment bovine genome graphs for different cattle breeds [65].

Using microarray-derived genotypes as a truth set may overestimate the accuracy of sequence variant discovery particularly at variants that are rare or located in less accessible regions of the genome. Moreover, it does not allow assessment of the accuracy and sensitivity of indel discovery because variants other than SNPs are currently not routinely genotyped with commercially available microarrays. Estimating the proportion of opposing homozygous genotypes between parent–offspring pairs may be a useful diagnostic metric to detect sequencing artefacts or flawed genotypes at indels [66]. Our results show that genotypes at indels are more accurate using *Graphtyper* than either *SAMtools* or *GATK* because *Graphtyper* produced less opposing homozygous genotypes at indels in nine sire-son pairs than the other methods both in the raw and filtered datasets. These findings are in line with those reported by Eggertsson et al. [32], who showed that the mapping of the sequencing reads to a variation-aware graph could improve read alignment nearby indels, thus enabling highly accurate sequence variant genotyping also for variants other than SNPs. Recently, Garrison et al. [34] showed that graph-based variant discovery may also mitigate reference allele bias. An assessment of reference allele bias was, however, not possible in our study because the sequencing depth was too low for most samples.

In our study, *Graphtyper* required less computing time than *GATK* to genotype sequence variants for 49 individuals. *SAMtools* required the least computing resources, probably because the implemented *mpileup* algorithm produces genotypes from the aligned reads without performing the computationally intensive local realignment of the reads. However, with an increasing number of samples, the multi-sample variant genotyping implementation of the *GATK HaplotypeCaller* module seems to be more efficient than *SAMtools mpileup* because variant discovery within samples can be separated from the joint genotyping across samples [19, 57]. A highly parallelized graph-based variant discovery pipeline also offers a computationally feasible and scalable framework for variant discovery in thousands of samples [32]. However, the computing time necessary for graph-based variant genotyping might be high in genomic regions where the nucleotide diversity is high or the assembly is flawed [35, 67]. In our study, the algorithm implemented in the *Graphtyper* software failed to finish within the allocated time for 12 1-Mb segments including a segment on chromosome 12 that contains a large segmental duplication [61, 68, 69] possibly because many mis-mapped reads increased graph complexity. The region on chromosome 12 contains an unusually large number of sequence variants and has been shown to suffer from low accuracy of imputation [61]. *Graphtyper* also failed to finish within the allocated time for a region on chromosome 23 that encompasses the bovine major histocompatibility complex, which is known to have a high level of diversity. Our results show that *Graphtyper* may also produce genotypes for problematic segments when they are split and processed in smaller parts. Moreover, most of these problems disappeared when we considered the latest assembly of the bovine genome, which possibly corroborates that more complete and contiguous genome assemblies may facilitate more reliable genotyping from variation-aware graphs [37, 70].

## Conclusions

Genome graphs facilitate sequence variant discovery from non-linear reference genomes. Sequence variant genotyping from a variation-aware graph is possible in cattle using *Graphtyper*. Sequence variant genotyping at both SNPs and indels is more accurate and sensitive using *Graphtyper* than either *SAMtools* or *GATK*. The proportion of Mendelian inconsistencies at both SNPs and indels is low using *Graphtyper*, which indicates that sequence variant genotyping from a variation-aware genome graph facilitates accurate variant discovery at different types of genetic variation. Considering highly informative variation-aware genome graphs that have been constructed from multiple breed-specific de-novo assemblies and high-confidence sequence variants may facilitate more accurate, sensitive and unbiased sequence variant genotyping in cattle.

## Additional files

**Additional file 1.** Instruction to compile a *Graphtyper* version modified for the cattle chromosome complement.

**Additional file 2.** Properties of the different metrics used for the evaluation of sequence variant genotyping accuracy. The metrics were calculated using the sum of the red cells as numerator and the cells within the green frame as denominator.

**Additional file 3.** Concordance of heterozygous and alternate homozygous genotypes in 49 Original Braunvieh cattle (a) and the concordance at the different sequencing depth for the (b) raw and (c) imputed datasets.

**Additional file 4.** Sequence variant genotyping quality for 18 and 31 animals that were sequenced at a lower and higher than 12-fold sequencing coverage, respectively. Asterisks denote significant differences (* *P* ≤ 0.05, ** *P* ≤ 0.01, *** *P* ≤ 0.001) with the best value (italic) for a respective parameter.

**Additional file 5.** Twelve 1-Mb regions for which *Graphtyper* initially failed to genotype sequence variants because the algorithm either ran out of memory or exceeded the allocated runtime (12 h). *Graphtyper* eventually produced genotypes for the sequence variants when these regions were re-run in 10-kb segments.

**Additional file 6.** Comparison of variant filtration using either *GATK* hard filtering or *GATK* VQSR.

**Additional file 7.** Accuracy and sensitivity of sequence variant genotyping on bovine chromosome 25 from a variation-aware genome graph that incorporated 2,143,417 dbSNP variants as prior known variants.

## Data Availability

The scripts for three pipelines are available via *GitHub* repository (https://github.com/danangcrysnanto/Graph-genotyping-paper-pipelines). Instructions to install a modified *Graphtyper* version for the bovine chromosome complement are provided in Additional file 1. Sequencing read data of 49 Original Braunvieh bulls are available from the European Nucleotide Archive (ENA) (http://www.ebi.ac.uk/ena) under primary accession PRJEB28191.
