# Supplementary figures and images for: Accurate sequence variant genotyping in cattle using variation-aware genome graphs
Source: Genet Sel Evol. 2019 May 15;51:21. doi: 10.1186/s12711-019-0462-x (PMC6521551; doi:10.1186/s12711-019-0462-x)

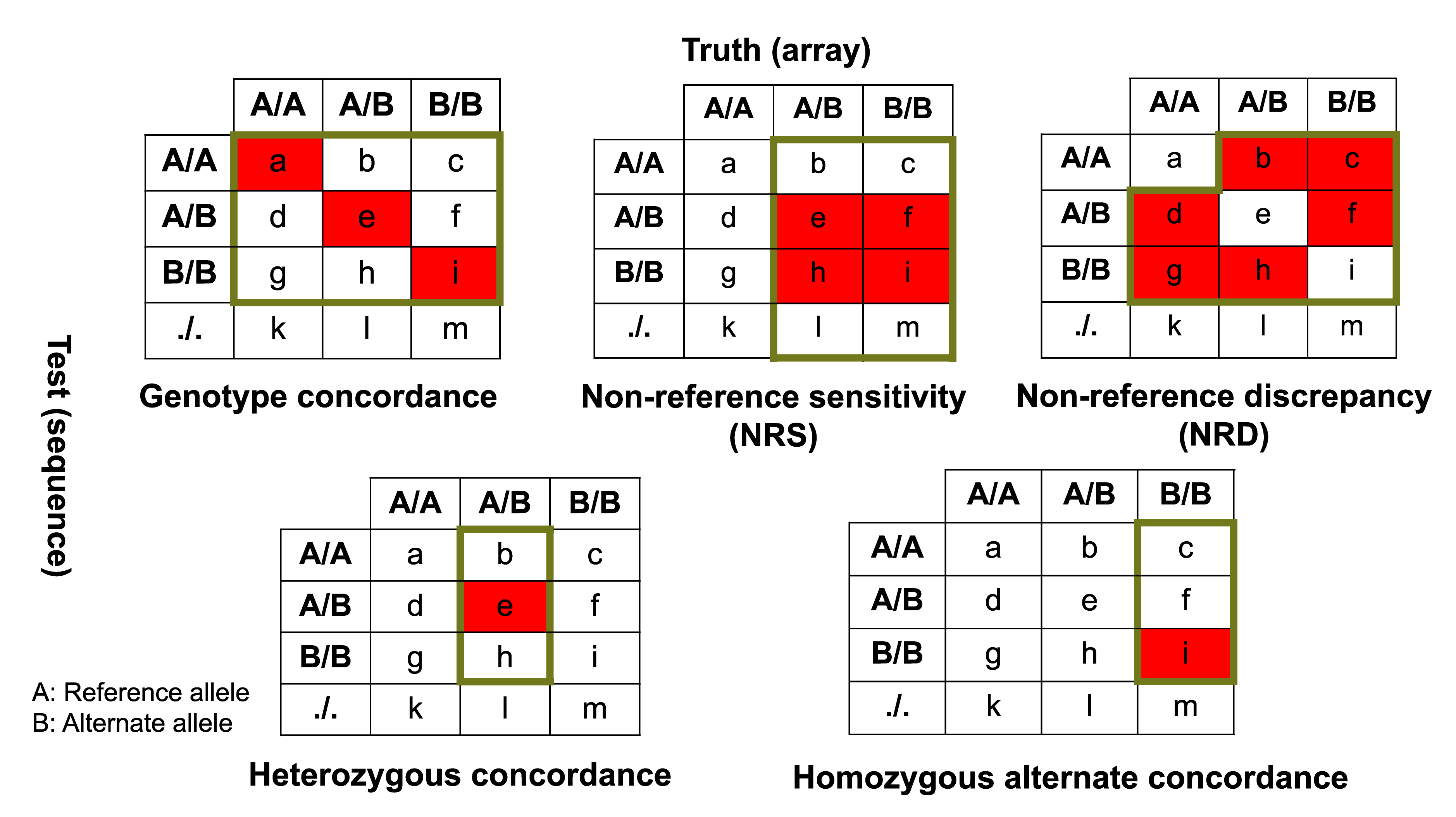

Supplement: Supplementary file 2 — Additional file 2. Properties of the different metrics used for the evaluation of sequence variant genotyping accuracy. The metrics were calculated using the sum of the red cells as numerator and the cells within the green frame as denominator. [file 12711_2019_462_MOESM2_ESM.tif]

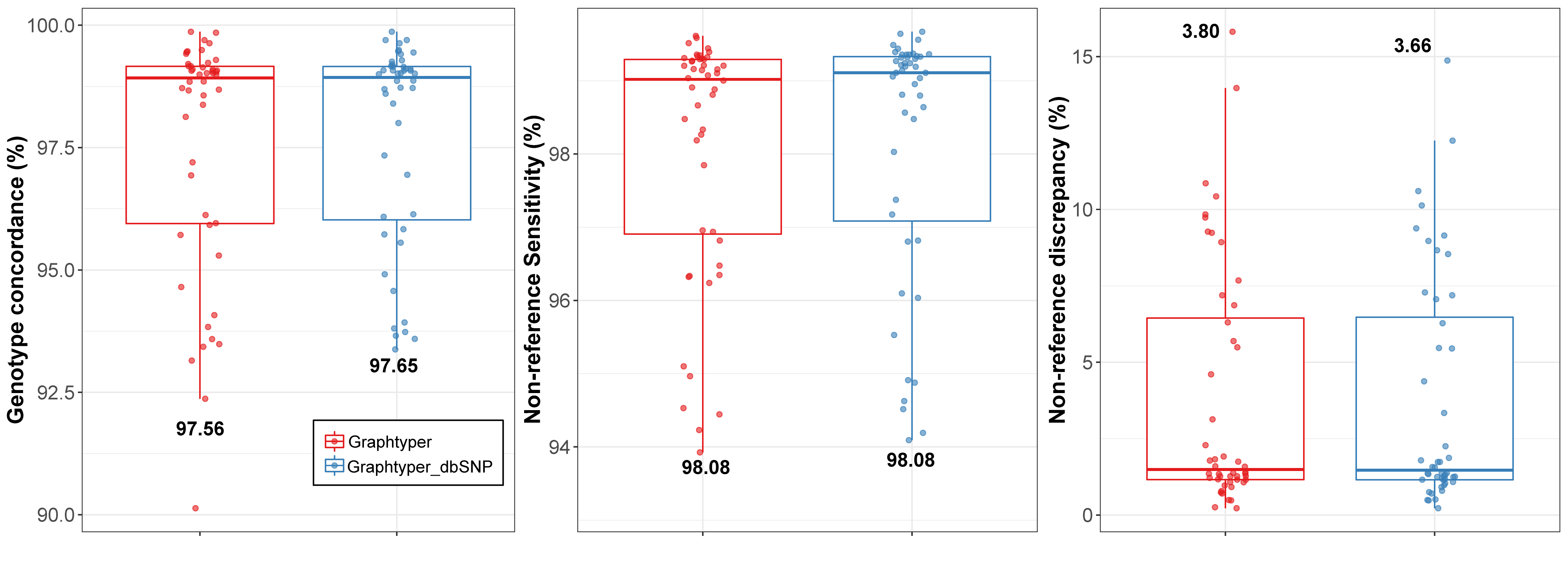

Supplement: Supplementary file 7 — Additional file 7. Accuracy and sensitivity of sequence variant genotyping on bovine chromosome 25 from a variation-aware genome graph that incorporated 2,143,417 dbSNP variants as prior known variants. [file 12711_2019_462_MOESM7_ESM.tif]
