# Supplementary material for: Accurate sequence variant genotyping in cattle using variation-aware genome graphs
Source: Genet Sel Evol. 2019 May 15;51:21. doi: 10.1186/s12711-019-0462-x (PMC6521551; doi:10.1186/s12711-019-0462-x)
Supplement: Supplementary file 3 — Additional file 3. Concordance of heterozygous and alternate homozygous genotypes in 49 Original Braunvieh cattle (a) and the concordance at the different sequencing depth for the (b) raw and (c) imputed datasets. [file 12711_2019_462_MOESM3_ESM.pdf]

a

	Heterozygous concordance				Homozygous concordance			
	full		filtered		full		filtered	
	raw	imp	raw	imp	raw	imp	raw	imp
GATK	89.17	99.11	89.24	99.21	*98.74*	99.18	*98.75*	99.27
Graphtyper	*95.79*	*99.36*	*95.82*	*99.44*	98.55	*99.51*	98.59	*99.57*
SAMtools	95.73	98.91	95.77	98.99	98.46	99.37	98.48	99.41

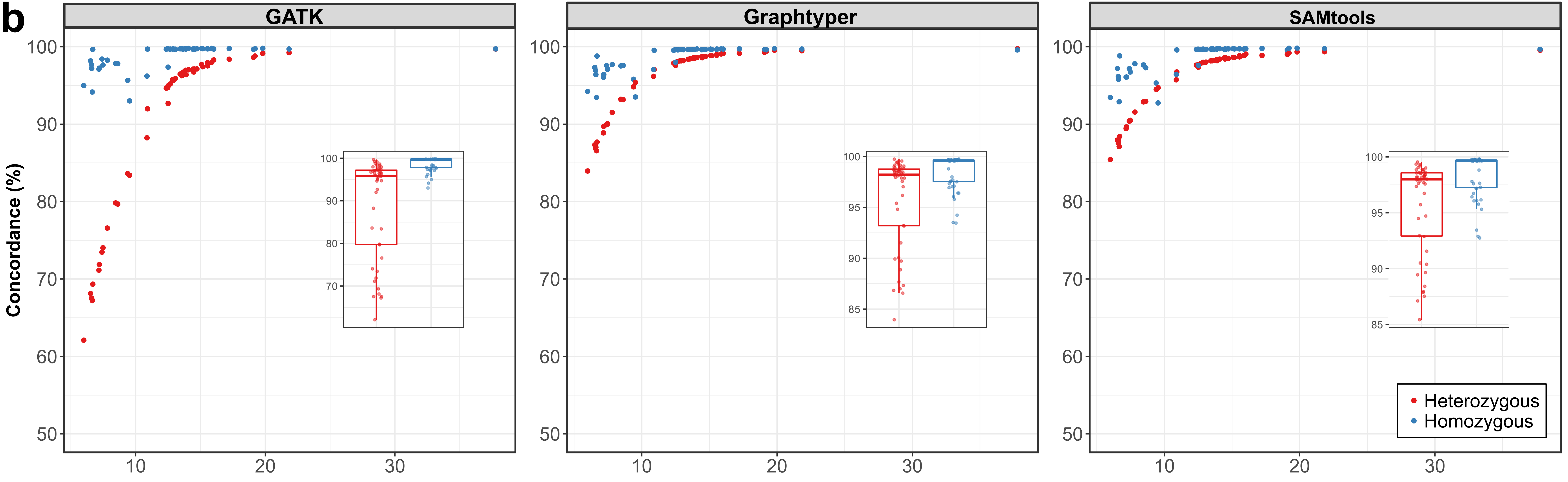

b
GATK
Graphtyper
SAMtools
Concordance (%)
Heterozygous
Homozygous

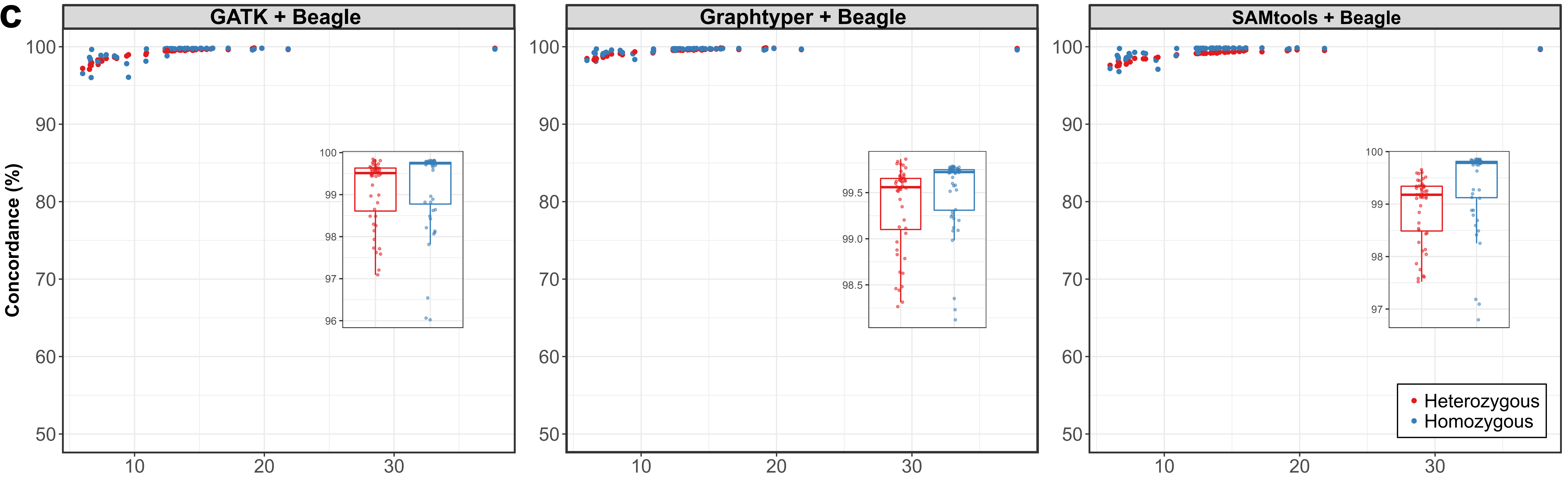
c
GATK + Beagle
Graphtyper + Beagle
SAMtools + Beagle
Concordance (%)
100
90
80
70
60
50
10
20
30
100
99
98
97
96
99.5
99.0
98.5
100
99
98
97
Heterozygous
Homozygous
