# Supplementary material for: Accurate sequence variant genotyping in cattle using variation-aware genome graphs
Source: Genet Sel Evol. 2019 May 15;51:21. doi: 10.1186/s12711-019-0462-x (PMC6521551; doi:10.1186/s12711-019-0462-x)
Supplement: Supplementary file 6 — Additional file 6. Comparison of variant filtration using either GATK hard filtering or GATK VQSR. [file 12711_2019_462_MOESM6_ESM.pdf]

<u>Supplementary note on variant filtration using *GATK*</u>

The best practice guidelines for variant discovery using *GATK* recommend sequence variants to be filtered using Variant Quality Score Recalibration (VQSR) because it implements advanced machine learning-based methods to differentiate between true and false-positive variants. However, VQSR relies on sets of high confidence truth/training variants, which are currently not (publicly) available in cattle. Thus, we ran *GATK* with best practice recommendations for variant filtering when applying VQSR is not possible, i.e., we used a generic baseline hard-filtering threshold for each variant annotation (see https://gatkforums.broadinstitute.org/GATK/discussion/2806/howto-apply-hard-filters-to-a-call-set). This threshold-based filtering is commonly applied the cattle genomics community (Koufariotis *et al.* 2018; Chen *et al.* 2018).

To facilitate running the VQSR module in sheep and goat, i.e., species where sets of truth/training variants are not (publicly) available, Alberto and colleagues (2018) used an intersection of high confidence variants that had been discovered from multiple variant callers as truth/training sets, i.e., they derived truth/training sets directly from the analyzed data. We implemented their approach to apply *GATK* VQSR to our variant dataset. Training and truth sets were constructed using the overlap of the filtered variants from the *GATK*, *Graphtyper* and *SAMtools* pipelines (truth=false, training=true, known=false, prior= 10) and markers from the BovineHD BeadChip (truth=true, training=true, known=false, prior= 15), respectively. Moreover, we used variants listed in dbSNP (version 150) as known variants (truth=false, training=false, known=true, prior=3.0). Following GATK VQSR, we retained variants in the 99.9% tranche sensitivity threshold (best practice).

Variant filtration using *GATK* VQSR removed more variants from the raw data than *GATK* hard filtering (Table 1). However, VQSR retained more HD SNPs than *GATK* hard filtering, possibly reflecting bias that results from the use of HD SNPs as training/truth sets. The values of the concordance statistics (genotype concordance, non-reference sensitivity, non-reference discrepancy) were almost identical between *GATK* VQSR and *GATK* hard filtration (Table 2) indicating that the choice of either filtration option does not notably affect the concordance between sequence-derived and BovineHD SNP array-derived genotypes. These findings are in line with Vander Jagt *et al.* (2018) who showed that the concordance between microarray-called and sequence-derived genotypes is almost identical using either *GATK* VQSR or the *GATK* 1000 bull genomes project hard filters, even though they used stringently filtered truth/training sets based on a more comprehensive catalogue of variants than in our study. Interestingly, in agreement with Vander Jagt *et al.* (2018), the proportion of opposing homozygous genotypes in sire/son-pairs (which does not suffer from ascertainment bias because it is calculated using sequence-derived SNPs) is less using *GATK* hard filter than *GATK* VQSR.

The performance of *GATK* VQSR may be assessed using the novel variant sensitivity tranche plot (Figure 2). In the lowest 90% tranches (highest specificity) the filtering model still retained many false positive variants (orange box and low Ti/Tv ratio). However, when the 99.9% tranche sensitivity is used as filtration criterion as recommended by the *GATK* best practice guidelines, a high proportion of true positive variants is removed from the data.

Overall, our findings suggest that
- (i) *GATK* VQSR removes more variants from the data than *GATK* hard filtering,
- (ii) *GATK* VQSR does not notably improve the concordance between sequence-derived and microarray-called genotypes compared to *GATK* hard filtering,
- (iii) the proportion of opposing homozygous genotypes in sire/son-pairs is higher using *GATK* VQSR than *GATK* hard filtering, and
- (iv) improving VQSR may be possible by providing more sophisticated truth/training variant datasets produced by orthogonal sequencing technology other than the ones used for training, e.g. (Li *et al.* 2018).

Table 1 Comparison of variants statistics between unfiltered and filtered datasets using either hard-filtering or VQSR.

	GATK full	*GATK* hard-filter	*GATK* VQSR
Total SNPs	18,594,182	17,248,593	16,537,577
Biallelic	18,347,962	17,111,806	16,430,734
Multi-allelic	246,220	136,787	106,843
Ti/Tv ratio	2.09	2.17	2.16
BovineHD	99.46	99.21	99.38
BovineSNP50	99.14	98.91	98.98

Table 2 The concordance statistics between hard-filtered and VQSR

	Genotype concordance	Non-reference sensitivity	Non-reference discrepancy	Opposing Homozygous
GATK hard-filter	96.02	93.67	6.3	0.72
GATK VQSR	96.01	93.77	6.32	0.75

Figure 1 Tranche sensitivity plot of novel variants as reported by the VQSR model fitting

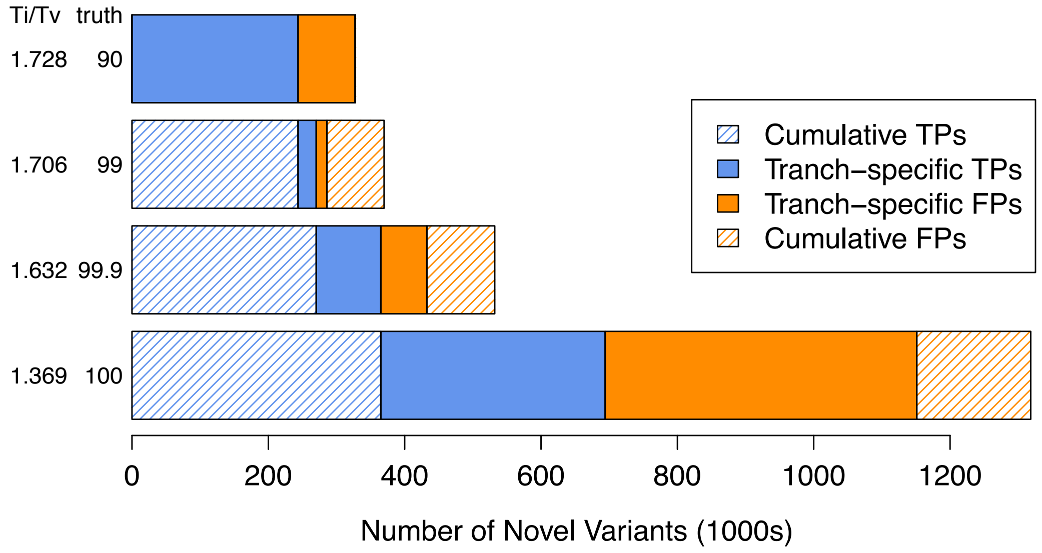

References

Alberto F. J., F. Boyer, P. Orozco-terWengel, I. Streeter, B. Servin, *et al.*, 2018 Convergent genomic signatures of domestication in sheep and goats. Nat. Commun. 9: 813. https://doi.org/10.1038/s41467-018-03206-y

Chen N., Y. Cai, Q. Chen, R. Li, K. Wang, *et al.*, 2018 Whole-genome resequencing reveals world-wide ancestry and adaptive introgression events of domesticated cattle in East Asia. Nat. Commun. 9: 2337. https://doi.org/10.1038/s41467-018-04737-0

Koufariotis L., B. J. Hayes, M. Kelly, B. M. Burns, R. Lyons, *et al.*, 2018 Sequencing the mosaic genome of Brahman cattle identifies historic and recent introgression including polled. Sci. Rep. 8: 17761. https://doi.org/10.1038/s41598-018-35698-5

Li H., J. M. Bloom, Y. Farjoun, M. Fleharty, L. Gauthier, *et al.*, 2018 A synthetic-diploid benchmark for accurate variant-calling evaluation. Nat. Methods 15: 595–597. https://doi.org/10.1038/s41592-018-0054-7

Vander Jagt CJ, Chamberlain AJ, Schnabel RD, Hayes BJ, Daetwyler HD. Which is the best variant caller for large whole-genome sequencing datasets?. Proc. 11th World Conr. Genet. Appl. Livest. Prod. (WCGALP), 11.128, Auckland, New Zealand, Accessed Feb. 1, 2019.
